# Evaluation of Anti-HIV-1 Integrase and Anti-Inflammatory Activities of Compounds from *Betula alnoides* Buch-Ham

**DOI:** 10.1155/2019/2573965

**Published:** 2019-06-02

**Authors:** Prapaporn Chaniad, Teeratad Sudsai, Abdi Wira Septama, Arnon Chukaew, Supinya Tewtrakul

**Affiliations:** ^1^School of Medicine, Walailak University, Nakhon Si Thammarat 80160, Thailand; ^2^Research Center for Chemistry, Indonesian Institute of Sciences, Kawasan Puspitek Serpong, Tangerang Selatan, Banten 15314, Indonesia; ^3^Chemistry Department, Faculty of Science and Technology, Suratthani Rajabhat University, Surat Thani 84100, Thailand; ^4^Faculty of Pharmaceutical Sciences, Prince of Songkla University, Hat-Yai, Songkhla 90112, Thailand

## Abstract

*Betula alnoides* is a medicinal plant in Thai traditional longevity preparations. The crude extracts of this plant possess various biological activities. However, the isolated compounds from this plant have no reports of anti-HIV-1 integrase (IN) activity. Therefore, the present study aims to investigate the anti-HIV-1 integrase and anti-inflammatory effects of isolated compounds from this plant and predict the interaction of compounds with integrase active sites. From the bioassay-guided fractionation of the ethanol extract of *B. alnoides* stems using chromatographic techniques, five pentacyclic triterpenoid compounds were obtained. They are betulinic acid (**1**), betulin (**2**), lupeol (**3**), oleanolic acid (**4**), and ursolic acid (**5**). Compound **2** exhibited the most potent inhibitory activity against HIV-1 IN, with an IC_50_ value of 17.7 *μ*M. Potential interactions of compounds with IN active sites were investigated using computational docking. The results indicated that active compounds interacted with Asp64, a residue participating in 3′-processing, and Thr66, His67, and Lys159, residues participating in strand-transfer reactions of the integration process. Regarding anti-inflammatory activity, all compounds exerted significant inhibitory effects on LPS-induced nitric oxide production (IC_50_ < 68.7 *μ*M). Thus, this research provides additional scientific support for the use of *B. alnoides* in traditional medicine for the treatment of HIV patients.

## 1. Introduction

Human immunodeficiency virus (HIV) infection remains a major global public health crisis. In 2017, there were approximately 36.9 million people living with HIV, with 1.8 million people becoming newly infected and 940,000 people died from HIV-related causes globally [1]. The infection leads to a progressive immunodeficiency due to the depletion of CD4+ T-cells and increased susceptibility to opportunistic infections as a result of their immunocompromised state [2]. HIV infection is also associated with a rapid and intense release of a variety of cytokines, which is associated with relatively high levels of inflammation [3]. Integration of transcribed viral DNA into the host chromosome is mediated by the integrase (IN) enzyme which is a key enzyme for viral integration of the reverse-transcribed viral DNA into the host cell genome, an essential step in the HIV life cycle [4]. The integration requires two catalytic reactions, referred to as 3′-processing and DNA strand transfer [5]. The full-length IN structure consists of three functional domains. The N-terminal domain, residues 1–51, contains a conserved HCCHZn^2+^-binding motif. The catalytic core domain, residues 52–210, contains the catalytic triad characterized by Asp64, Asp116, and Glu152. The C-terminal domain, residues 220–288, contributes to DNA binding [6]. Currently, only three IN inhibitors, i.e., raltegravir, elvitegravir, and dolutegravir, have been approved by the FDA [7]. However, these drugs have limited clinical benefit because long-term treatments may lead to the emergence of drug resistance and side effects [8]. Therefore, finding agents from natural products is an alternative approach for novel HIV-1 inhibitors with high selectivity and low toxicity.

*Betula alnoides* (Betulaceae family) is locally known in Thai as “Khamlang suea khrong.” The stem bark of this plant has traditionally been used for tonic, longevity, and appetite and as a carminative and an aphrodisiac. Methanol and ethanol extracts of this plant possess various biological activities, such as anti-inflammatory [9], anti-hyperlipidemia, anti-oxidant, anti-microbial, *α*-glucosidase inhibitory activities [10], and anti-diabetic effects [11]. Our preliminary screening of Thai traditional medicine used as agents assisting longevity revealed that the water and ethanol extract of *Betula alnoides* wood possessed high inhibitory activity against HIV-1 IN with an IC_50_ of 10.2 and 20.1 *μ*g/mL [12]. It is important to note that there have been no reports describing any anti-HIV-1 IN activity of isolated compounds from this plant. Therefore, the aims of this study are to isolate pure compounds, evaluate their anti-HIV-1 IN and anti-inflammatory activities, and predict the potential interactions of the compounds with HIV-1 IN using a molecular docking technique.

## 2. Materials and Methods

### 2.1. Plant Materials

*B. alnoides* stems were collected from Chonburi Province, Thailand, in 2015 and were identified by a traditional Thai doctor, Mr. Sarupsin Thongnoppakhun. The voucher specimen (SKP024020101) was deposited at the Department of Pharmacognosy and Pharmaceutical Botany, Faculty of Pharmaceutical Sciences, Prince of Songkla University, Thailand.

### 2.2. General Experimental Procedure

The NMR spectra were recorded in CDCl_3_ on a Varian Unity Inova at 500 MHz for ^1^H and 125 MHz for ^13^C (chemical shifts in *δ*, ppm). Column chromatography was performed using silica gel (230–400 mesh, SiliCycle Inc., Canada), Sephadex LH-20, and Diaion HP-20 (Sigma-Aldrich, USA). All solvents were analytical reagent grade and purchased from Labscan, Thailand. All reagents were purchased from Sigma, USA.

### 2.3. Extraction and Isolation of Compounds

The dried coarse powder of *B. alnoides* stems (800 g) was extracted three times with 95% ethanol under reflux for 3 h. The filtrate was concentrated at 50°C under reduced pressure to obtain ethanol extract (83.9 g). This extract was subsequently partitioned with various solvents to generate residues of hexane (7.2 g), chloroform (21.5 g), ethyl acetate (15.3 g), water (25.4 g), and water and chloroform emulsion (10.3 g) fractions. These fractions were prepared at concentrations 3–100 *μ*g/mL for screening of their anti-HIV-1 IN activity.

The water and chloroform fractions that exhibited good activity with IC_50_ values of 20.5 and 25.5 *μ*g/mL, respectively (Table 1), were further isolated to obtain the pure compounds. The water fraction (15.0 g) was applied to a Diaion HP-20 column and eluted by a step gradient starting with water, mixtures of water and methanol, and then mixtures of methanol and ethyl acetate to obtain six pooled major fractions (W1–W6), based on TLC analysis. Fraction W3 (3.2 g) was further isolated by vacuum liquid chromatography (VLC) with chloroform and increasing polarity with methanol as the eluent to give compound **1** (200.9 mg, 1.139% w/w) as white needle crystals.

The chloroform fraction (12.5 g) was chromatographed by VLC using silica gel. Elution was started with hexane and chloroform and followed by ethyl acetate and methanol to give four fractions (C1–C4). Fraction C1 (4.1 g) was chromatographed over silica gel and eluted with chloroform and increasing polarity with ethyl acetate to obtain compound **2** (38.6 mg, 0.309% w/w) as a white powder. Fraction C2 (3.3 g) was chromatographed by VLC using chloroform and increasing polarity with ethyl acetate and methanol as the eluent to give 5 subfractions (C2/1–C2/5). Subfraction C2/2 was rechromatographed on silica gel to afford compound **3** (15.6 mg, 0.124% w/w) as a white powder. Fractions C3 (2.5 g) and C4 (3.8 g) were purified by the same procedure, successively affording compounds **4** (15.6 mg, 0.030% w/w) and **5** (8.1 mg, 0.064% w/w) as white powder, respectively.

The structures of compounds **1**–**5** were identified by ^1^H and ^13^C-NMR analysis as well as by comparison with previously reported data in the literature.

### 2.4. Assay of HIV-1 IN Inhibitory Activity

The anti-HIV IN activity of isolated compounds was determined in an *in vitro* model using HIV-1 IN enzymes according to the multiplate integration assay (MIA) as previously described [13]. Briefly, a mixture (45 *μ*L) composed of 12 *μ*L of IN buffer (containing 150 mM 3-(*N*-morpholino)propanesulfonic acid, pH 7.2 (MOPS), 75 mM MnCl_2_, 5 mM dithiothreitol (DTT), 25% glycerol, and 500 *μ*g/mL bovine serum albumin), 1 *μ*L of 5 pmol/mL digoxigenin-labeled target DNA, and 32 *μ*L of sterilized water was added into each well of a 96-well plate. Subsequently, 6 *μ*L of sample solution in DMSO and 9 *μ*L of a 1/5 dilution of the IN enzyme were added to each well and incubated at 37°C for 80 min. After washing the plate three times with PBS with 0.05% Tween 20 (PBST), 100 *μ*L of 500 mU/mL alkaline phosphatase- (AP-) labeled anti-digoxigenin antibody was added and incubated at 37°C for 1 h. The plates were washed with PBS three times. Then, AP buffer (150 *μ*L) containing 100 mM Tris-HCl (pH 9.5), 100 mM NaCl, 5 mM MgCl_2_, and 10 mM *p*-nitrophenyl phosphate was added to each well and incubated at 37°C for 1 h. Finally, the absorbance of *p*-nitrophenol, the final product of the integration reaction, was measured with a microplate reader (Rayto, RT-2100C) at a wavelength of 405 nm. Suramin, a polyanionic HIV-1 IN inhibitor, was used as a positive control.

### 2.5. Assay of Anti-Inflammatory Activity

To evaluate the anti-inflammatory activity, an inhibitory effect on nitric oxide (NO) production was carried out according to the previous report described by Sudsai et al. [14]. Briefly, RAW264.7 cells were seeded onto 96-well plates (1 × 10^5^ cells/well) and were maintained to adhere at 37°C for 1 h in a CO_2_ incubator containing 5% CO_2_. They were then cultured in RPMI-1640 medium containing lipopolysaccharide (LPS, 100 ng/ml) together with the test compounds at various concentrations (3–100 *μ*M). After 24 h of incubation, the nitrite (NO^2–^) concentration in the culture medium was determined as an indicator of NO production using the Griess reagent to assay the accumulation of NO^2–^, a stable metabolite of NO. The absorbance was measured using a microplate reader at 570 nm. In this study, NO synthase inhibitor (L-nitro-arginine, L-NA), nuclear translocation of NF-*κ*B inhibitor (caffeic acid phenethyl ester, CAPE) and nonsteroidal anti-inflammatory drug, NSAID (indomethacin), were used as positive controls. The percent inhibition was calculated from the following equation, and inhibition concentration at 50% (IC_50_) values was determined graphically (*n* = 4):

(1) $$\begin{array}{c} \text{Inhibition }\left(\%\right)\;=\;\left[\frac{\left(A-B\right)}{\left(A-C\right)}\right]\times 100, \end{array}$$

where *A–C* are the NO^2–^ concentration (*A* = LPS (+), sample (−); *B* = LPS (+), sample (+); *C* = LPS (−), sample (−)).

### 2.6. Viability Assay of RAW264.7 Macrophage Cells

The cytotoxicity of the test compounds after 24 h of incubation was determined by the colorimetric method described by Sudsai et al. [14]. A volume of 10 *μ*l of MTT solution (5 mg/ml in PBS) was added to each well of 96-well plates and further incubated in a CO_2_ incubator for 4 h. The formazan products generated by MTT reduction were dissolved in DMSO. At last, the medium was removed, 100 *μ*l of DMSO was then added to each well and thoroughly mixed by gently tapping on the test plate. The absorbance of formazan solution was measured at a wavelength of 570 nm using a microplate reader. The test compounds were considered to be cytotoxic when the viability of the compound-treated group was less than 80% of that in the control (1% DMSO-treated) group.

### 2.7. Molecular Docking Method

Molecular docking experiments of HIV-1 IN enzyme and pure compound were performed with version 4.2 of the AutoDock program according to the procedure as previously described [15]. Docking calculations were carried out using the Lamarckian genetic algorithm (LGA) with 100 docking runs for each ligand to explore the best conformational space. An initial population size was set at 150 randomly placed individuals. The maximum number of energy evaluations was increased to 2,500,000 per run, and the genetic generation was 100,000. The lowest binding energy-docked conformation of the most populated cluster was chosen for analysis of the H-bond interactions.

### 2.8. Statistical Analysis

The results are expressed as the mean value ± S.E.M. of four determinations. Differences between groups were assessed by one-way ANOVA using the post hoc Duncan's test. The significance level was considered at *p* < 0.05.

## 3. Results

### 3.1. Extraction and Isolation of Compounds

**Figure 1:**
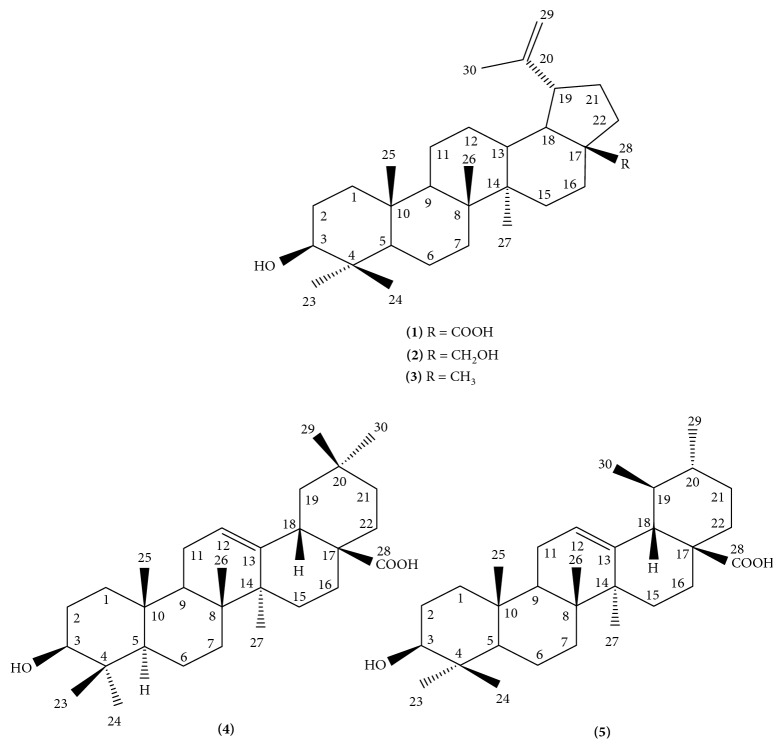
The structures of compounds isolated from *B. alnoides* (**1**: betulinic acid; **2**: betulin; **3**: lupeol; **4**: oleanolic acid; **5**: ursolic acid).

From bioassay-guided fractionation based on anti-HIV-1 IN activity using the MIA method, the bioactive water and chloroform fractions were purified by chromatographic techniques to afford five known pentacyclic triterpenoid compounds (Figure 1). They were identified as three lupane-type compounds: betulinic acid, **1** [16, 17]; betulin, **2** [18]; and lupeol, **3** [19], along with one oleanane-type compound, oleanolic acid, **4** [17], and one ursane-type compound, ursolic acid, **5** [20].

#### 3.1.1. Betulinic Acid (**1**): White Crystal Needle (200.9 mg)

^1^H-NMR (CDCl_3_): *δ* 3.18 (1H, *dd*, *J* = 4.8 Hz, H-3), 2.98 (1H, *m*, H-19), 4.56 (1H, *dd*, *J* = 2.0, 1.5 Hz, H-29a), 4.71 (1H, *d*, *J* = 2.0 Hz, H-29b), 0.91, (3H, *s*, H-23), 0.75 (3H, *s*, H-24), 0.83 (3H, *s*, H-25), 0.94^*∗*^(3H, *s*, H-26), 0.96^*∗*^ (3H, *s*, H-27), 1.63 (3H, *s*, H-30). ^*∗*^Interchangeable signals.

^13^C-NMR (CDCl_3_): *δ* 38.6 (C-1), 27.3 (C-2), 78.8 (C-3), 38.6 (C-4), 55.5 (C-5), 18.3 (C-6), 34.0 (C-7), 40.4 (C-8), 50.5 (C-9), 37.7 (C-10), 20.8 (C-11), 25.5 (C-12), 38.4 (C-13), 42.4 (C-14), 30.5 (C-15), 32.1 (C-16), 56.3 (C-17), 46.9 (C-18), 49.3 (C-19), 150.4 (C-20), 39.7 (C-21), 37.0 (C-22), 28.2 (C-23), 15.3 (C-24), 15.9 (C-25), 16.1 (C-26), 14.5 (C-27), 179.7 (C-28), 109.6 (C-29), 19.4 (C-30).

#### 3.1.2. Betulin (**2**): White Powder (38.6 mg)

^1^H NMR (CDCl_3_): *δ* 4.62 (1H, *d*, *J* = 2.2, H29b), 4.54 (1H, *dd*, *J* = 2.0, 1.5 Hz, H-29a), 3.78 (1H, *d*, *J* = 10.9, H-28b), 3.31 (1H, *d*, *J* = 10.9, H-28a), 3.16 (1H, *dd*, *J* = 11.4, 4.6, H-3), 1.66 (3H, *s*, H-30), 0.96 (3H, *s*, H-27), 0.99 (3H, *s*, H-26), 0.95 (3H, *s*, H-23), 0.80 (3H, *s*, H-25), 0.74 (3H, *s*, H-24).

^13^C NMR (CDCl_3_): *δ* 150.5 (C20), 109.7 (C-29), 79.0 (C-3), 60.5 (C-28), 55.3 (C-5), 50.4 (C-9), 48.7 (C-18), 47.9 (C-17), 47.8 (C-19), 42.7 (C-14), 40.9 (C-8), 38.8 (C-4), 38.7 (C-1), 37.3 (C-13), 37.1 (C-10), 34.2 (C-7), 34.0 (C-22), 29.7 (C-21), 29.1 (C-16), 28.0 (C-23), 27.4 (C-2), 27.0 (C-15), 25.2 (C-12), 20.9 (C-11), 19.1 (C-30), 18.3 (C-6), 16.1 (C-25), 16.0 (C-26), 15.3 (C-24), 14.7 (C-27).

#### 3.1.3. Lupeol (**3**): White Powder (15.6 mg)

^1^H NMR (CDCl_3_): *δ* 4.68 (1H, *d*, *J* = 2.4 Hz, H-29a), 4.55 (1H, *dd*, *J* = 2.4, 1.4 Hz, H-29a), 3.20 (1H, *dd*, *J* = 11.4, 4.7 Hz, H-3), 1.66 (3H, *s*, H-30), 0.92 (3H, *s*, H-27), 1.01 (3H, *s*, H-26), 0.95 (3H, *s*, H-23), 0.85 (3H, *s*, H-25), 0.79 (3H, *s*, H-28), 0.74 (3H, *s*, H-24).

^13^C NMR (CDCl_3_): *δ* 151.0 (C-20), 109.3 (C-29), 79.0 (C-3), 55.5 (C-5), 50.5 (C-9), 48.3 (C-18), 48.0 (C-19), 43.0 (C-17), 42.9 (C-14), 40.8 (C-8), 40.1 (C-22), 39.0 (C-13), 38.9 (C-4), 38.6 (C-1), 37.2 (C-10), 35.6 (C-16), 34.3 (C-7), 29.9 (C-21), 28.0 (C-23), 27.4 (C-15), 27.5 (C-12), 24.4 (C-2), 20.9 (C-11), 19.3 (C-30), 18.5 (C-6), 18.1 (C-28), 16.2 (C-25), 16.0 (C-26), 15.6 (C-24), 14.5 (C-27).

#### 3.1.4. Oleanolic Acid (**4**): White Powder (8.1 mg)

^1^H NMR (CDCl_3_): 2.82 (1H, *m*, H-18), 2.87 (1H, *m*, H-19), 3.23 (1H, *dd*, *J* = 11.0, 4.8 Hz, H-3), 5.27 (1H, *dd*, *J* = 3.8, 3.6 Hz, H-12), 0.80 (3H, *s*, H-26), 1.05 (3H, *s*, H-23), 0.95 (3H, *s*, H-30), 0.93 (3H, *s*, H-25), 0.91 (3H, *s*, H-29), 0.80^*∗*^ (3H, *s*, H-26), 0.79^*∗*^ (3H, *s*, H-24). ^*∗*^interchangeable signals.

^13^C NMR (CDCl_3_): *δ* 180.1 (C-28), 143.6 (C-13), 123.0 (C-12), 79.0 (C-3), 55.2 (C-5), 48.0 (C-9), 46.6 (C-19), 46.5 (C-17), 42.4 (C-18), 41.8 (C-14), 39.5 (C-8), 39.1 (C-1), 38.9 (C-4), 37.1 (C-10), 33.9 (C-21), 33.5 (C-29), 32.8 (C-7), 33.1 (C-22), 31.1 (C-20), 28.4 (C-23), 28.1 (C-2), 27.8 (C-15), 26.4 (C-27), 23.8 (C-30), 23.8 (C-11), 23.6 (C-16), 18.8 (C-6), 17.2 (C-26), 16.9 (C-24), 15.8 (C-25).

#### 3.1.5. Ursolic Acid (**5**): White Powder (22.4 mg)

^1^H NMR (CDCl_3_): *δ* 5.25 (1H, *dd*, *J* = 3.7, 3.4 Hz, H-12), 3.25 (1H, *dd*, *J* = 10.8, 5.1 Hz, H-3), 1.00 (1H, *m*, H-19), 1.05 (3H, *s*, H-27), 0.98 (3H, *d*, *J* = 6.5 Hz, H-30), 1.10 (3H, *s*, H-23), 0.95 (3H, *s*, H-25), 0.88 (3H, *d*, *J* = 6.5 Hz, H-29), 0.79 (3H, *s*, H-24), 0.83 (3H, *s*, H-26).

^13^C NMR (CDCl_3_): *δ* 179.7 (C-28), 138.2 (C-13), 126.0 (C-12), 78.8 (C-3), 55.3 (C-5), 53.9 (C-18), 48.2 (C-17), 47.5 (C-9), 42.1 (C-14), 39.6 (C-8), 39.1 (C-19), 39.0 (C-20), 38.7 (C-1), 38.6 (C-4), 37.6 (C-22), 37.2 (C-10), 33.0 (C-7), 30.2 (C-21), 29.0 (C-23), 28.8 (C-15), 28.0 (C-2), 25.1 (C-16), 23.8 (C-11), 23.6 (C-27), 21.5 (C-30), 18.3 (C-6), 17.2 (C-26), 17.0 (C-29), 15.7 (C-24), 15.5 (C-25).

All isolated compounds are known triterpenoids that are found in many plants, especially in birch species (*Betula* spp.), and exhibited a wide spectrum of biological and pharmacological activities. However, it is important to note that these compounds have not previously been investigated for anti-HIV-1 IN activity. In addition, the anti-inflammatory activity of *B. alnoides* has been only reported in methanol and ethanol extracts. Therefore, all identified compounds were evaluated for anti-HIV-1 IN effect as well as anti-inflammatory activity.

### 3.2. HIV-1 IN Inhibitory Activity

The results revealed that betulin (**2**) is the most potent anti-HIV-1 IN activity with an IC_50_ value of 17.7 *μ*M. Betulinic acid (**1**) showed good inhibition of HIV-1 IN with an IC_50_ value of 24.8 *μ*M. However, oleanolic acid (**4**) and ursolic acid (**5**) showed moderate activity with IC_50_ values of 30.3 and 35.0 *μ*M, respectively, whereas lupeol (3) was inactive against HIV-1 IN (Table 2).

### 3.3. Anti-Inflammatory Activity

All compounds exhibited different degrees of anti-inflammatory effects in a concentration-dependent manner (Table 3). Betulin and betulinic acid possessed good activity with IC_50_ values of 30.1 and 31.0 *μ*M, respectively. Lupeol showed moderate activity with an IC_50_ value of 47.3 *μ*M, while oleanolic acid and ursolic acid exhibited weak activity with IC_50_ values of 62.8 and 68.7 *μ*M, respectively. Remarkably, betulin and lupeol showed significant NO suppression and caused cytotoxicity to RAW 264.7 cells.

### 3.4. Molecular Docking

**Figure 2:**
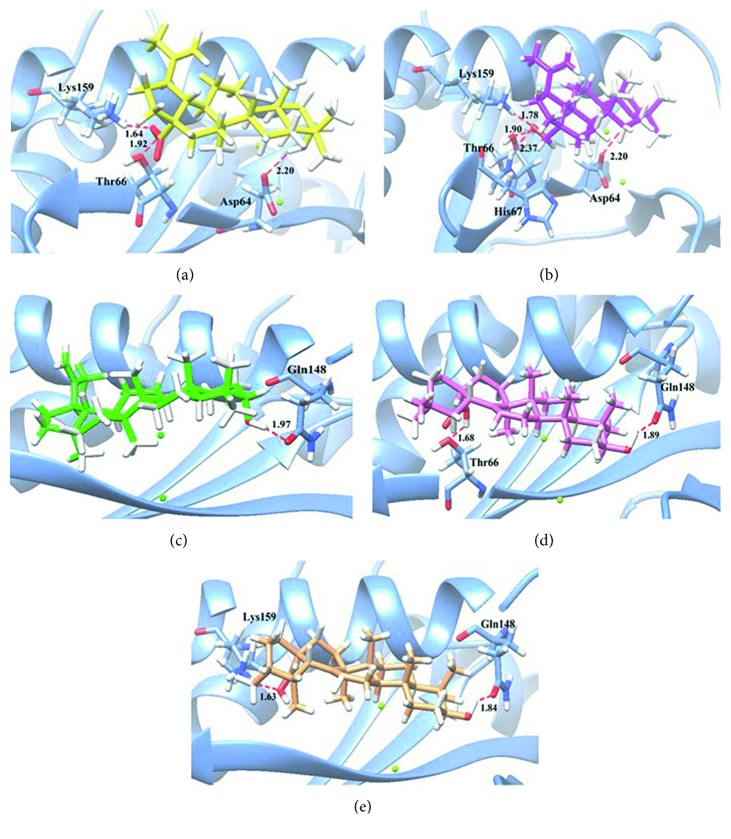
Molecular docking of the isolated compounds with HIV-1 IN. The ribbon model shows the backbone of the HIV-1 IN catalytic domain with all interacting amino acid residues shown as stick models and colored by heteroatoms. H-bond interactions are shown as red dashed lines and represent bond length in angstroms (Å). Mg^2+^ ions are shown as green balls. (a) Betulinic acid (**1**), (b) betulin (**2**), (c) lupeol (**3**), (d) oleanolic acid (**4**), and (e) ursolic acid (**5**).

The interactions of compounds with the amino acid residues of IN are shown in Figure 2, and the docking results are summarized in Table 4. The results showed that betulin (**2**) possessed the best binding affinity for the IN enzyme in terms of low binding energy (−5.75 kcal/mol) and lowest inhibiting constants (*K*_i_, 72.26 *μ*M), indicating that it strongly interacted with IN. Betulin exhibited four hydrogen bond interactions with amino acid residues. The hydroxyl group at position C-28 interacted with Asp64, the residue of the catalytic triad, while the hydroxyl group at C-3 formed multiple hydrogen bonds with Thr66, His67, and Lys159. Betulinic acid (**1**) interacted with Asp64, Thr66, and Lys159. The binding energy of betulinic acid was lower than that of betulin (**2**). Lupeol (**3**), which contained a methyl group at position C-17, was an inactive compound against HIV-1 IN. It only interacted with Gln148 and had a weak binding energy (−3.28 kcal/mol). Oleanolic acid (**4**) and ursolic acid (**5**) formed two hydrogen bonds with weak interactions that can be observed in terms of binding energy.

**Figure 3:**
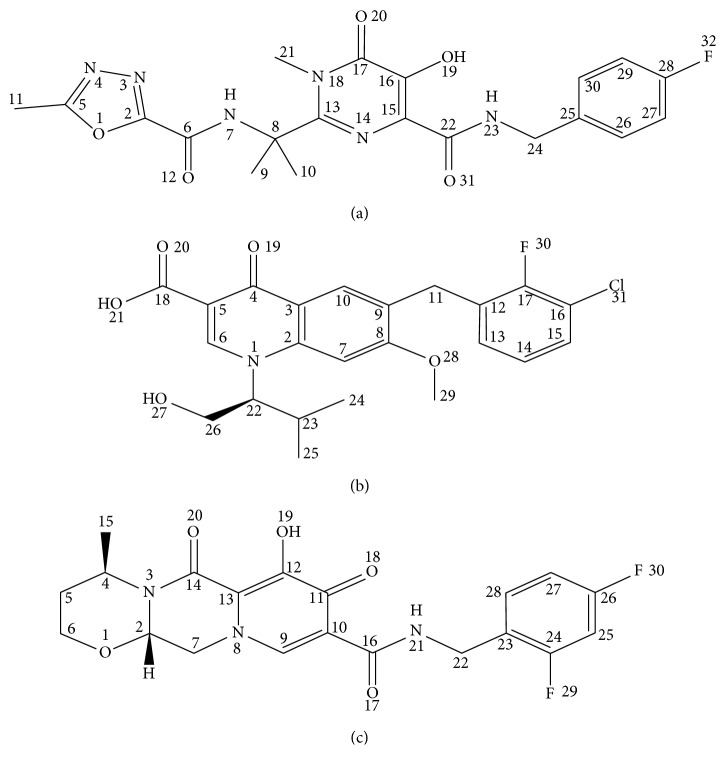
The structures of HIV-1 IN inhibitors. (a) Raltegravir, (b) elvitegravir, and (c) dolutegravir.

**Figure 4:**
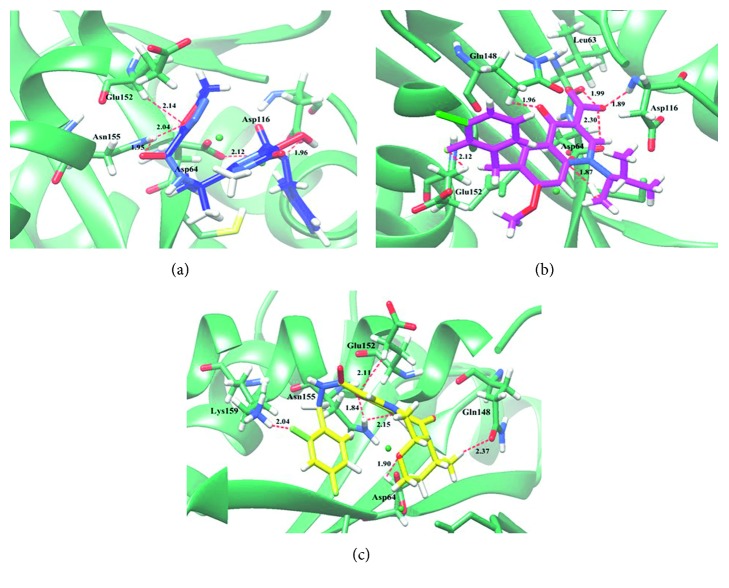
Molecular docking of HIV-1 IN inhibitors with HIV-1 IN. The ribbon model shows the backbone of the HIV-1 IN catalytic domain with all interacting amino acid residues shown as stick models and colored by heteroatoms. H-bond interactions are shown as red dashed lines and represent bond length in angstroms (Å). Mg^2+^ ions are shown as green balls. (a) Raltegravir, (b) elvitegravir, and (c) dolutegravir.

In this study, the potential interactions of drugs as IN inhibitors (Figure 3) with HIV-1 IN enzyme were also investigated using the molecular docking technique. The result showed that raltegravir, elvitegravir, and dolutegravir strongly interacted with IN with binding energies of −6.98, −7.10 and −6.51 kcal/mol, respectively, and formed with different amino acids (Table 5). The predicted binding interaction of these inhibitors within the HIV-1 IN active site are illustrated in Figure 4. Raltegravir interacted with all catalytic triad residues of IN, including Asp64, Asp116, and Glu152 as well as Asn155. Elvitegravir possessed the lowest *K*_i_ value (5.25 *μ*M) and exhibited lowest binding energy. In addition, it formed six H-bonding with Leu63, Asp64, Asp116, Gln148, and Glu152. In the case of dolutegravir, it formed comparable numbers of H-bonding to elvitegravir but showed weaker interaction with the enzyme than elvitegravir in terms of high binding energy and *K*_i_ value (17.62 *μ*M).

## 4. Discussion

The structure of betulin, the compound that possessed the strongest activity, has three remarkable positions, the primary hydroxyl group at position C-28, the secondary hydroxyl group at position C-3, and the alkene moiety at position C-20. In the case of betulinic acid, the structure is substituted with a carboxylic group at C-17. It possessed less activity than betulin, which was confirmed by weaker interactions with the IN active site in terms of the lower number of hydrogen bonds. With respect to lupeol, the structure was substituted with a methyl group in the same position, and lupeol had considerably decreased activity against HIV-1 IN. These results agree with previous reports that found lupeol was poorly active for antiviral activity [21]. Interestingly, the docking result does correlate well with their activity, in which there is a relationship between the binding energy, number of hydrogen bonds, and potency against HIV-1 IN.

These results clearly show the structure-activity relationship that minor structural modifications at C-17 of those pentacyclic triterpenoids lead to significant differences in the inhibitory anti-HIV-I IN effect. In particular, hydroxyl groups are a potential functional group for binding to IN active sites, resulting in the inhibitory action against IN. Asp64, Thr66, and His67 are amino acid residues participating in 3′-processing, and Gln148, Asn155, and Lys159 are residues participating in strand-transfer reactions. Thus, this result underlined that the anti-HIV-1 IN activity of active compounds resulted from interference with the integration process at the IN active site. Docking studies of three IN inhibitors revealed that all inhibitors strongly interacted with amino acid residue of IN enzyme. All inhibitors are found to bind preferably in similar ways close to the catalytic residues, Asp64, Asp116, and Glu152. Their binding energy and *K*_i_ show that IN inhibitors interact more strongly with HIV-1 IN than isolated compound from *B. alnoides*. The oxadiazole group of raltegravir is an essential function group to interact with HIV-1 IN active site. In addition, the halobenzyl groups of elvitegravir and dolutegravir display the important role for interaction.

Regarding the anti-inflammatory activity, our study is in accordance with previous studies in which triterpenoid compounds presented anti-inflammatory effects in various models. Betulin, the compound that possessed highest activity in this study, also exhibited an anti-inflammatory effect by the reduction of NO level in the edema paw model [22]. The potential of betulinic acid to exert anti-inflammatory activity was supported by a study conducted by Viji et al. [23] that it inhibited the cyclooxygenase 2 (COX-2) expression in cell cultures and also reported to protect the mice against lipopolysaccharide (LPS) by modulating tumor necrosis factor *α* (TNF-*α*) production [24]. For lupeol, a previous report has shown that this compound decreased TNF-*α* and interleukin *β* (IL*β*) in LPS-treated macrophages [25] as well as shown to decrease the level of cytokines IL-4, IL-5, and IL-13 in a bronchial asthma mouse model [26]. In the case of oleanolic acid, it was observed to significantly inhibit the activity of acetic acid-induced hyperpermeability and carboxymethylcellulose-induced leukocyte migration *in vivo* which mediated by the downregulation of the expression of NF-*κ*B and TNF-*α* production [27]. Ursolic acid was reported to reduce the levels of IL-1*β*, IL-6, and TNF-*α* and to increase the production of IL-10 in macrophages stimulated with LPS.

Since pentacyclic triterpenes are secondary metabolites widespread in various plants, betulinic acid and betulin are lupane-type triterpenes which can be found in large amount in the outer bark of many species of birch, i.e., *Betula pendula* Roth, *B. pubescens* Ehrh, and *B. davurica* Pall [28–30]. For betulinic acid, it was also the most prominent secondary metabolite present in the fruit of *Dillenia indica* which is extensively used as a food additive [31] and has been previously isolated from the stems of *Combretum laxum* [17]. Moreover, it was also isolated from aerial parts of *Euphorbia microsciadia* [32], stem bark of *Syzygium guineense* Wild DC [33], and *Polypodium vulgare,* the common polypody, is a fern widely distributed in Europe [34]. Betulin has been found predominantly in the bark of birch trees and various plants, including *Acacia mellifera* [35], *Byrsonima microphylla* [36], the twigs of *Celtis philippinensis* [37], and stem bark of *Adenium obesum* [18]. Lupeol was isolated from *Acacia mellifera* [35] and *Chrysanthemum indicum* Linne [38]. This compound has also been found in *Polypodium vulgare* [34]. In particular, lupeol, oleanolic acid, and ursolic acid were found in the flower part of *Gentiana veitchiorum* [39]. In addition, ursolic acid and oleanolic acid were isolated from the leaves of *Orthosiphon stamineus* [40] and the leaves of *Perilla frutescens* var. *acuta* [41].

In terms of effect of medicinal plants and constituents against HIV-IN, several medicinal plants have been described as possessing anti-HIV-1 IN activity. Our previous study showed that the crude ethanolic and aqueous extracts from eight plants of Thai medicinal plants in longevity preparations; *Albizia procera*, *Areca catechu*, *Bauhinia strychnifolia*, *Betula alnoides*, *Blumea balsamifera*, *Caesalpinia sappan*, *Cassia garrettiana*, and *Stephania venosa* possess good activity with IC_50_ values of <20 *μ*g/ml [12]. Regarding isolated compounds exhibiting an effect against HIV-1 IN, luteolin and luteolin 7-methyl ether isolated from *Coleus parvifolius* have been reported to be potent anti-HIV-1 agents [42]. (+)-Catechin was isolated from *Albizia procera* bark exhibited appreciable activity against HIV-1 IN with an IC_50_ value of 46.3 *μ*M, whereas protocatechuic acid showed mild activity with 46.0% inhibition at a concentration of 100 *μ*M [43]. The heartwoods and roots of *Caesalpinia sappan* L. was phytochemically investigated by Tewtrakul et al. [44], and the results revealed that sappanchalcone displayed the strongest effect against HIV-1 IN with an IC_50_ value of 2.3 *μ*M followed by protosappanin A with an IC_50_ value of 12.6 *μ*M. For the compounds isolated from the bulbils *of Dioscorea bulbifera*, myricetin exhibited the most potent activity with an IC_50_ value of 3.15 *μ*M, followed by 2,4,6,7-tetrahydroxy 9,10-dihydrophenanthrene IC_50_ value of 14.20 *μ*M [45]. The active compound, *N*-methyl-trans-4-hydroxy-L-proline was isolated from *Aglaia andamanica* leaves. It has been reported to be potent anti-HIV-1 agents with an IC_50_ value of 11.8 *μ*g/mL [46]. In addition, bisdemethoxycurcumin from the rhizomes of *Boesenbergia kingii* showed moderate anti-HIV-1 IN with an IC_50_ value of 47.7 *μ*M [47].

Regarding the other biological activities of isolated compounds, betulin has been reported to possess antiviral [48] and anticancer activities [49]. Betulinic acid exhibited anti-HIV-1 reverse transcriptase activity [50], antimalarial [51], anticancer [52], and antibacterial effects [53]. Lupeol, oleanolic acid, and ursolic acid have shown anti-inflammatory and anticancer properties [54, 55].

## 5. Conclusions

Pentacyclic triterpenoids were isolated from the stems of *B. alnoides*, including betulinic acid (**1**), betulin (**2**), oleanolic acid (**4**), and ursolic acid (**5**). These compounds showed significant anti-HIV activity with IC_50_ values ranging from 17.7 to 35.0 *μ*M and possessed anti-inflammatory effects. The active compounds against HIV-1 IN interacted with the essential amino acids participating in 3′-processing and strand-transfer reactions, resulting in interference with the integration process. This finding is the first report of the anti-HIV-1 IN activity of compounds from *B. alnoides*.

## Figures and Tables

**Table 1:** Anti-HIV-1 IN activity of *B. alnoides* extract and its fractions.

Sample	IC_50_ (*μ*g/mL)
Ethanol extract	17.6 ± 1.5^b^
*n*-Hexane fraction	>100
Chloroform fraction	25.5 ± 1.4^d^
Ethyl acetate fraction	76.5 ± 1.7^e^
Water fraction	20.5 ± 0.7^c^
Emulsion of water and chloroform fraction	>100
Suramin (positive control)	3.9 ± 0.3^a^

Each value represents mean ± S.E.M. of four determinations. Different characters (a, b, c, d, and e) indicate significant differences among the compared means which in the same treatment group at *p* < 0.05.

**Table 2:** Anti-HIV-1 integrase activity of pure compounds from *B. alnoides*.

Sample	% Inhibition at various concentrations (*μ*M)	IC_50_ (*μ*M)				
		1	3	10	30	100
Betulinic acid (**1**)	—	—	39.0 ± 2.3	53.2 ± 1.2	65.6 ± 2.1	24.8 ± 1.3^c^
Betulin (**2**)	—	—	41.2 ± 1.5	57.6 ± 1.2	70.7 ± 2.1	17.7 ± 0.6^b^
Lupeol (**3**)	—	—	12.2 ± 1.3	25.9 ± 2.0	40.6 ± 1.8	>100
Oleanolic acid (**4**)	—	—	33.4 ± 1.8	49.1 ± 2.2	65.5 ± 1.5	30.3 ± 1.3^d^
Ursolic acid (**5**)	—	—	31.6 ± 0.9	48.5 ± 1.2	60.2 ± 2.1	35.0 ± 1.0^e^
Suramin	32.1 ± 0.6	50.3 ± 1.6	70.9 ± 1.5	93.3 ± 1.3	99.9 ± 1.7	2.8 ± 0.1^a^

Each value represents the mean ± S.E.M. of four determinations. Different characters (a, b, c, d, and e) indicate significant differences among the compared means in the same treatment group at *p* < 0.05.

**Table 3:** Anti-inflammatory activity of isolated compounds from *B. alnoides* in RAW264.7 cells.

Sample	% Inhibition at various concentration (*μ*M)	IC_50_ (*μ*M)				
		0	3	10	30	100
Betulinic acid (**1**)	0.0 ± 3.4	14.6 ± 2.3	22.1 ± 1.2	49.4 ± 3.2	79.5 ± 1.7	31.0 ± 1.9^b^
Betulin (**2**)	0.0 ± 3.5	2.9 ± 1.9	13.0 ± 1.6	58.0 ± 1.1^*∗*^	81.8 ± 1.2^*∗*^	30.1 ± 0.3^b^
Lupeol (**3**)	0.0 ± 3.5	6.0 ± 1.7	11.4 ± 2.7	23.4 ± 1.9^*∗*^	78.2 ± 1.0^*∗*^	47.3 ± 1.0^c^
Oleanolic acid (**4**)	0.0 ± 3.5	10.9 ± 1.8	18.4 ± 1.2	28.2 ± 3.2	63.4 ± 1.0	62.8 ± 3.6^d^
Ursolic acid (**5**)	0.0 ± 3.5	5.4 ± 2.3	10.2 ± 3.2	13.6 ± 3.2	67.1 ± 1.8	68.7 ± 5.9^d^
L-NA	0.0 ± 3.4	19.3 ± 4.7	22.1 ± 1.9	32.6 ± 3.5	79.7 ± 4.8	40.4 ± 6.1^bc^
Indomethacin	0.0 ± 3.4	32.0 ± 2.6	43.8 ± 1.2	54.1 ± 2.0	79.0 ± 1.4	17.6 ± 1.6^a^
CAPE	0.0 ± 3.4	22.7 ± 3.1	48.6 ± 0.7	66.8 ± 0.5	85.9 ± 1.0	12.3 ± 0.8^a^

Each value represents the mean ± S.E.M. of four determinations. Different characters (a, b, c, and d) indicate significant differences among the compared means in the same treatment group at *p* < 0.05.

**Table 4:** Molecular docking results of pure compounds from *B. alnoides*.

Compounds	Lowest binding energy (kcal/mol)	*K*_i_	Amino acid	H-bond interaction	Distance (Å)
Betulinic acid (**1**)	−5.36	118.36 *μ*M	Asp64	OD2---3-HO	2.20
			Thr66	OG1---28-HO	1.92
			Lys159	HZ3---28-OH	1.64

Betulin (**2**)	−5.75	72.26 *μ*M	Asp64	OD2---28-HOCH_2_	2.20
			Thr66	OG1---3-HO	1.90
			His67	HN---3-OH	2.37
			Lys159	HZ3---3-OH	1.78

Lupeol (**3**)	−3.28	3.95 mM	Gln148	OE1---3-HO	1.97

Oleanolic acid (**4**)	−3.53	2.59 mM	Thr66	OG1---28-HO	1.68
			Gln148	OE1---3-HO	1.89

Ursolic acid (**5**)	−3.68	1.52 mM	Gln148	OE1---3-HO	1.84
			Lys159	HZ3---28-OH	1.63

**Table 5:** Molecular docking study of drugs as HIV-IN inhibitors.

Integrase inhibitors	Lowest binding energy (kcal/mol)	*K*_i_ (*μ*M)	Amino acid	H-bond interaction	Distance (Å)
Raltegravir	−6.98	7.68	Asp64	OD1----23HN	2.12
			Asp116	OD2----23HN	1.96
			Glu152	HA----12O	2.14
			Asn155	HD21----12O	2.04
			Asn155	H21----1O	1.95

Elvitegravir	−7.20	5.25	Leu63	O----21HO	1.99
			Asp64	OD2----27HO	1.87
			Asp64	OD1----21HO	1.89
			Asp116	HN----27OH	1.89
			Gln148	HG2----19O	1.96
			Glu152	HB1----30F	2.12

Dolutegravir	−6.51	17.62	Asp64	HB2----1O	1.90
			Gln148	OE1----15H_3_C	2.37
			Glu152	HG2----18O	2.11
			Asn155	HD21----18O	1.84
			Asn155	HD21----19OH	2.15
			Lys159	HD23----29F	2.04

## Data Availability

The data used to support the findings of this study are available from the corresponding author upon request.
